# Consumption of Ultra-Processed Foods and Sustainable Lifestyles: A Multicenter Study

**DOI:** 10.3390/nu18020365

**Published:** 2026-01-22

**Authors:** Eliana Romina Meza-Miranda, Solange Parra-Soto, Leslie Landaeta-Díaz, Israel Rios-Castillo, Patricio Pérez-Armijo, Tannia Valeria Carpio-Arias, Macarena Jara Nercasseau, Georgina Gómez, Brian M. Cavagnari, Jacqueline Araneda-Flores, Karla Cordón-Arrivilaga, Catalina Ramirez-Contreras, Carla Villagran-Cerro, Ana Gabriela Murillo, Gladys Morales, Melissa Miranda-Durán, Ana María Aguilar, Alfonsina Ortiz, Edna J. Nava-González, Jhon Jairo Bejarano-Roncancio, Beatriz Núñez-Martínez, João P. M. Lima, Jorge de Assis Costa, Jairo Torres, Saby Mauricio, Saby Camacho, Gloria Maricela Morales, Samuel Durán-Agüero

**Affiliations:** 1 Centro Multidisciplinario de Investigaciones Tecnológicas, Universidad Nacional de Asunción, Campus San Lorenzo, San Lorenzo 2160, Paraguay; mezamirandaelianaromina@gmail.com; 2 Departamento de Nutrición y Salud Pública, Facultad Ciencias de la Salud y de los Alimentos, Universidad del Bío-Bío, Chillán 3780000, Chile; sparra@ubiobio.cl (S.P.-S.); jaraneda@ubiobio.cl (J.A.-F.); ctramirez@ubiobio.cl (C.R.-C.); carla.villagran1801@alumnos.ubiobio.cl (C.V.-C.); 3 Escuela de Nutrición y Dietética, Facultad de Salud y Ciencias Sociales, Universidad de Las Américas, Santiago 8330015, Chile; llandaeta@udla.cl; 4 Oficina Subregional de la FAO en Mesoamérica, Organización de las Naciones Unidas Para la Alimentación y la Agricultura (FAO), Panamá City 0843-00006, Panama; israel.rios@fao.org; 5 Facultad de Ciencias de la Salud, Universidad Isabel I, 09003 Burgos, Spain; patricioesteban.perez@ui1.es; 6 Grupo de Investigación en Alimentación y Nutrición Humana (GIANH), Facultad de Salud Pública, Escuela Superior Politécnica de Chimborazo, Riobamba 060104, Ecuador; tannia.carpio@espoch.edu.ec; 7 Escuela de Nutrición y Dietética, Facultad de Ciencias de la Rehabilitación y Calidad de Vida, Universidad San Sebastian, Providencia 7500000, Chile; mjaran@docente.uss.cl; 8 Departamento de Bioquímica, Escuela de Medicina, Universidad de Costa Rica, Montes de Oca, San José 2060, Costa Rica; georginagomez.ucr@gmail.com (G.G.); gmurillo.nutricion@gmail.com (A.G.M.); 9 Facultad de Ciencias Médicas, Pontificia Universidad Católica Argentina, Av. Alicia Moreau de Justo 1300, Buenos Aires C1107, Argentina; bcavagna@gmail.com; 10 Unidad de Investigación en Seguridad Alimentaria y Nutricional, Escuela de Nutrición, Facultad de Ciencias Químicas y Farmacia, Universidad de San Carlos de Guatemala, Guatemala City 01012, Guatemala; krcordon@gmail.com; 11 Departamento Salud Pública, Facultad de Medicina, Universidad de La Frontera, Temuco 4780000, Chile; gladys.morales@ufrontera.cl; 12 Instituto de Investigación en Salud y Desarrollo, Universidad Mayor de San Andrés, La Paz 02015, Bolivia; melm2m16@gmail.com (M.M.-D.); ana.aguilar@umsalud.edu.bo (A.M.A.); 13 Licenciatura en Nutrición, Facultad de Ciencias de la Salud, Universidad Católica del Uruguay, Montevideo 11600, Uruguay; alortiz@ucu.edu.uy; 14 Facultad de Salud Pública y Nutrición, Universidad Autónoma de Nuevo León, Monterrey 64460, Nuevo León, Mexico; edna.navagn@uanl.edu.mx; 15 Departamento de Nutrición Humana, Facultad de Medicina, Universidad Nacional de Colombia, Bogotá 111321, Colombia; jjbejaranor@unal.edu.co; 16 Coordinación General de Investigación, Universidad del Norte, Asunción 001218, Paraguay; beatrizelizabeth.85@gmail.com; 17 Health & Technology Research Center (H&TRC), Coimbra Health School, Polytechnic University of Coimbra, 3045-043 Coimbra, Portugal; joao.lima@estesc.ipc.pt; 18 Departamento de Ciências Humanas e Linguagens (DCHL), Universidade do Estado de Minas Gerais (UEMG), Núcleo de Estudo e Pesquisa em Educação e Saúde (NEPES), Ubá 36500-000, Brazil; nyron32@gmail.com; 19 FoodChemPack Research Group, Department of Analytical Chemistry, Nutrition and Food Science, Faculty of Pharmacy, University of Santiago de Compostela, Campus Vida, 15782 Santiago de Compostela, Spain; jairoalonsot@gmail.com; 20 Programa Académico Nutrición y Dietética, Facultad de Ciencias de la Salud, Universidad Privada Norbert Wiener, Lima 15000, Peru; saby.mauricio@gmail.com; 21 Dirección de Ciencias de la Salud, Facultad de Ciencias de la Salud, Universidad Latinoamericana, Lottus Education, Ciudad de México 14380, Mexico; saby.camacho@ula.edu.mx; 22 Instituto Salvadoreño de Bienestar Magisterial (ISBM), Policlínico de San Salvador y Centro de Hemodiálisis de El Salvador, San Salvador 503, El Salvador; gloria.morales@isbm.gob.sv

**Keywords:** ultra-processed foods, sustainable lifestyles, NOVA classification, Latin America

## Abstract

**Background:** Ultra-processed food (UPF) consumption has increased significantly in Latin America and Spain, impacting both health and environmental sustainability. To our knowledge, this is the first multicenter study to examine the association between UPF consumption and sustainable lifestyle behaviors in Latin America and Spain. **Objective:** To evaluate the association between UPF consumption and sustainable lifestyle behaviors in Latin America and Spain. **Methods:** This was an observational, analytical, multicenter, cross-sectional study. A validated, self-administered online questionnaire was distributed in 14 countries between March 2023 and January 2024. The survey collected sociodemographic data, UPF intake (classified using the NOVA system), body mass index and sustainable lifestyle behaviors (food, transport, environment). Multivariate linear regression models were applied to assess associations, adjusting for age, sex, smoking, physical activity and BMI. **Results:** Among 6009 adults (mean age: 34.98 ± 12.55; 79.5% women), those with the highest consumption of UPF (fast food, beverages and juices, salty snacks and sweet snacks) were significantly more likely to be in the least sustainable quartile compared to those who did not consume these food products ((OR = 2.51; 95% CI: 1.79–3.54), (OR = 1.82; 95% CI: 1.50–2.22), (OR = 1.51; 95% CI: 1.32–1.73) and (OR = 1.42; 95% CI: 1.20–1.67), respectively, with *p* values < 0.001). **Conclusions:** High consumption of ultra-processed foods (UPFs) is inversely associated with sustainable lifestyles. These findings position UPF consumption not only as a health problem but also as a key indicator of unsustainable lifestyles.

## 1. Introduction

Global rates of overweight and obesity have increased considerably over the past 40 years [1,2], leading researchers to explore the mechanisms that contribute to this phenomenon. While numerous biological (e.g., genetic and hormonal) and behavioral (e.g., sedentary lifestyle) factors are known to be associated with obesity [3], one of the key systemic changes in our environment, which has followed the rise in obesity rates, has been the exponential increase in ultra-processed foods (UPFs) in our food system since the 1980s [4,5]. UPFs are not found in nature and have been developed to improve the organoleptic characteristics, safety and shelf life of foods, among other purposes, by adding fats, sugars, salt and non-caloric sweeteners [6]. Some examples of UPF are packaged snacks (sweet and salty), fast food and sugary beverages [5,6]. Many of these products, while they may contain certain nutrients, have an intrinsic nutritional imbalance, compared to unprocessed foods, positioning them as products of low nutritional quality and with a potential negative impact on health. And although some may be fortified, they generally contain low levels of fiber, vitamins or minerals, to mention a few of their nutritional deficiencies [7].

Latin America and Spain share common cultural and dietary patterns and are undergoing similar nutritional transition processes. However, these regions have experienced rapid dietary changes driven by urbanization, market globalization and the aggressive marketing of packaged foods. The food price crisis has further contributed to poor diet quality, food insecurity and malnutrition [8]. While the association between ultra-processed food consumption and non-communicable diseases (NCDs) is well established, less is known about how these consumption patterns relate to sustainable lifestyle behaviors, including dietary, transport and environmental practices. Understanding this relationship is essential for designing integrated interventions that promote both health and sustainability [9,10].

In this sense, a sustainable lifestyle is considered to be a set of consumption patterns and habits that minimize negative environmental impact. The growing body of scientific evidence on sustainability increasingly highlights the importance of transforming food systems; therefore, there is currently more talk of sustainable diets than individual dietary prescriptions [11]. However, the current challenge is to address measurement aspects with indicator options that allow the assessment of sustainable lifestyles related to food in all its facets [12].

At the regional level, various studies have analyzed the consumption of UPF, finding high consumption in various age groups such as adults, university students, children and adolescents [13,14,15,16,17]. The results of this research highlight that excessive consumption of UPF is associated with middle to high incomes, obesity, lower quality of life and lower quality of sleep [18].

Furthermore, other regional studies have shown that UPF consumption is detrimental to the consumption of foods that are considered healthy; associated with increased blood glucose levels; negatively associated with sleep quality and overall diet quality; and associated with weight gain and consequently high rates of overweight and obesity, as well as increased consumption of fried foods [19,20,21,22,23].

There is growing evidence of the impact of our food choices on health and the environment [24]. Furthermore, the current production and consumption patterns of UPFs are estimated to contribute substantially to total greenhouse gas emissions and waste generation, and are considered a major cause of biodiversity loss, deforestation, water extraction and pollution [25,26]. Given that they are not nutritionally essential, are associated with adverse health effects, and generate significant economic and social costs, reducing their consumption could simultaneously benefit public health and environmental sustainability [27,28,29,30]. Nevertheless, existing reviews do not consider the diversity of terms used to classify foods such as UPFs, which can influence the interpretation of their environmental impacts in studies [31].

Recently, our research group at the Latin American Food and Nutrition Network (RedLIAN) found that people with plant-based diets showed a positive association with sustainable lifestyles, while people with Western dietary patterns showed a negative association, meaning that they had less sustainable lifestyles [32]. Despite the growing evidence linking UPF consumption to adverse health outcomes, its relationship with sustainable lifestyle behaviors remains largely unexplored. Addressing this gap is essential to inform integrated public health and sustainability strategies, particularly in regions undergoing rapid nutrition transitions.

Our hypothesis is that people who consume a high amount of UPF have lower sustainable lifestyle scores than those who consume less. Therefore, the objective of this study is to estimate the association of the frequency of UPF consumption with sustainable lifestyles in Latin America and Spain.

## 2. Materials and Methods

An observational, analytical, multicenter, cross-sectional survey-based study was conducted with a total of 6009 participants. The study took place between March 2023 and January 2024. The questionnaire was disseminated through social media (Instagram, Facebook, LinkedIn and X) and used non-probability snowball sampling.

### 2.1. Study Population

The final sample of the study was taken by convenience, where those who met the selection criteria were included as participants.

The inclusion criteria for participants were being 18 years of age or older, of both sexes, and residing in Latin America or Spain. Individuals with specific dietary patterns for medical reasons were excluded, for example, those with kidney or liver failure, or those receiving enteral nutrition.

A self-administered validated questionnaire was developed in an online format using the Google Docs interface. The questionnaire was divided into the following sections:

(1) Sociodemographic background: country of residence, age, sex, educational level, socioeconomic aspects and place of residence.
(2) Body mass index classification: BMI (kg/m^2^) was determined according to self-perceived weight (in kilograms) and height (in centimeters). For adult BMI classification: underweight < 18.5 kg/m^2^, normal 18.5 to <25 kg/m^2^, overweight 25 to <30 kg/m^2^, obese ≥ 30 kg/m^2^ [32].
(3) Ultra-processed foods: A validated survey on dietary habits was administered [33]. To assess the intake of selected ultra-processed foods, three questions on unhealthy foods were used to evaluate the consumption of sugary drinks, salty snacks and sweets; for example, “Do you consume sugary drinks or juices? (serving: 200 cc glass): (a) Does not consume; (b) Less than once a day; (c) 1 serving a day; (d) 2 servings a day; (e) 3 servings a day”. Based on these 5 response options, an overall classification was made into three categories: does not consume, moderate consumption (occasionally/week) and excessive consumption (1 serving/day; 2 servings/day; and equal to or greater than 3 servings/day). For the purposes of this research, moderate consumption was defined as the consumption of less than one serving per day, based on recommendations described in the literature indicating that consumption should be limited and only occasional [7].
(4) Sustainable Lifestyles Survey: This survey is subdivided into three items: the first consists of 15 questions assessing diet and shopping; the second, 12 questions assessing transportation, recreation and self-care; and the third, 11 questions assessing the environment. The total score for the Sustainable Lifestyles Survey is calculated as a continuous variable, with higher scores indicating more sustainable behaviors [31].

This survey was previously developed and validated in a recent study published by our research group, by experts in nutrition and public health [31]. All the questions in the questionnaire were accepted, and minimal modifications were made to the wording based on the experts’ suggestions, which proved to be appropriate in improving the clarity and understanding of the questionnaire in each country.

### 2.2. Ethical Aspects

The study was approved by the Ethics Committee of San Sebastián University (code 25-23). Upon opening the survey link, the informed consent form was presented, and upon agreeing to participate, the questions were displayed.

### 2.3. Universe and Sample

The sample size calculation was performed considering the data from the last national census of each country. Based on this detail, and considering a 90% confidence level, a sample of 271 participants was estimated for each country, which should be proportional to its population so that the weight of individuals is the same and each country has an equivalent representation for individuals over 18 years of age; only Uruguay presented a sample size below this threshold. This estimation was made using the GRANMO Grandária Mostral Calculator (https://www.datarus.eu/aplicaciones/granmo/, accessed on 6 June 2025).

### 2.4. Data Analysis

Statistical analyses were performed using the Stata 18.0 MP software (StataCorp, College Station, TX, USA). Quantitative variables are presented as means with standard deviations, and qualitative variables as frequencies and percentages. To evaluate associations between categorical variables, the Chi square test was used, along with one-way ANOVA for continuous variables in contingency tables with a significance of *p* < 0.05.

Multivariate logistic regression models were used to estimate associations between dietary patterns and sustainability scores. We evaluated key model assumptions, including the normality of residuals, homoscedasticity and multicollinearity. Given the large sample size, we assumed approximate normality based on the Central Limit Theorem. However, visual inspection of residual plots indicated potential heteroscedasticity. Therefore, we applied robust standard errors to account for non-constant variance. Multicollinearity was assessed using variance inflation factors, and no issues were identified.

All associations are reported as Odds Ratio (OR); 0 is the highest quartile with the most sustainable lifestyle score (high sustainable); 1 is quartiles 3, 2 and 1, with people with lower sustainable lifestyle scores (low sustainable), with their corresponding 95% confidence intervals (95% CIs) derived from models using robust standard errors, and a *p* value < 0.05 was considered indicative of statistical significance. Multivariate models were applied to adjust for country, sex, age, smoking, physical activity, residence and BMI.

## 3. Results

Overall, most of the study participants were female (78.5%) and younger than 60 years (95.2%), with an average age of 34.98 ± 12.55 years, and had a high school educational level (70.6%). The overall mean BMI was 25.30 ± 5.20 kg/m^2^. Among those with higher UPF intake, the BMI was significantly higher than among those with lower intake (25.91 ± 6.22 kg/m^2^). Furthermore, it was found that those with higher UPF intake were significantly younger (˂30 years). Furthermore, among subjects with a higher educational level, most of them had lower or no UPF intake. Furthermore, it can be noted that residents of rural areas have significantly lower UPF consumption than residents of urban areas. In almost all countries, residents of each of them predominantly consumed UPF moderately (Table 1).

**Figure 1:**
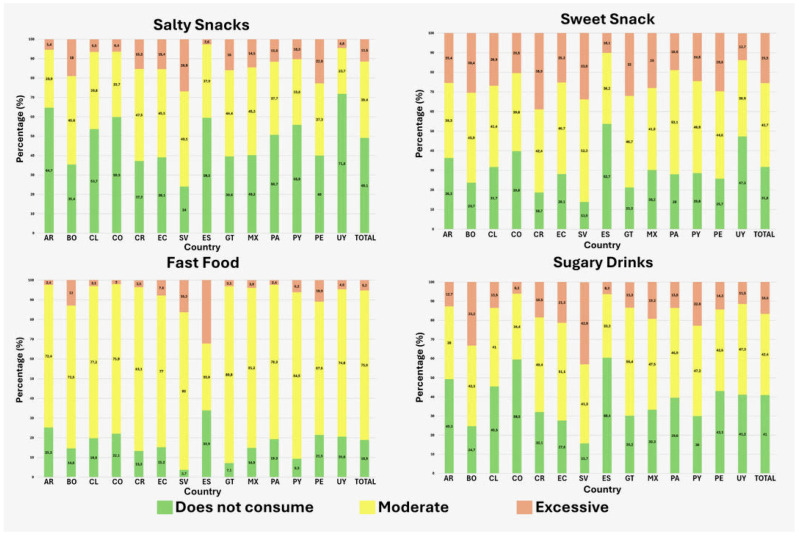
Distribution of ultra-processed food group consumption by country. AR: Argentina; BO: Bolivia; Cl: Chile; CO: Colombia; CR: Costa Rica; EC: Ecuador; SV: El Salvador; ES: Spain; GT: Guatemala; MX: Mexico; PA: Panama; PY: Paraguay; PE: Perú; UY: Uruguay.

Figure 1 shows the analysis of ultra-processed food (UPF) consumption by food group and country, revealing that El Salvador, Peru and Bolivia have the highest consumption of salty snacks, with percentages of 26.9%, 22.8% and 19%, respectively. Costa Rica, El Salvador and Guatemala had the highest consumption of sweet snacks (38.9%, 33.9% and 32.0% respectively). The countries with the highest fast food consumption profile were Guatemala, Paraguay and Costa Rica (89.8%, 84.5% and 83.1%, respectively). Regarding beverages and juices, El Salvador and Bolivia had the highest consumption (42.9% and 33.2%, respectively).

In general, moderate consumption was most prevalent in all food groups (Supplementary Table S1).

According to sociodemographic variables, it was observed that occasional fast food consumption was higher among those with basic education (78.6%), those younger than 30 years of age (79.7%) and women (76.4%). Beverages and juices were consumed mostly occasionally, specifically by people with a basic education level (46.3%), those younger than 30 years of age (47.4%) and men (43.4%). Sweet snacks were also consumed mostly occasionally by those with basic education (46.3%), those younger than 30 years of age (46.3%) and women (42.7%). On the other hand, it is highlighted that the consumption of salty snacks was lower in people with a medium academic level (51.4%), in those under 60 years of age (68.9%) and in women (50.3%) (Table 2).

Compared with non-consumers, moderate fast food consumption was associated with 75% higher odds of having a worse SLE (OR = 1.97; 95% CI: 1.70–2.29; *p* < 0.001), while daily (excessive) consumption almost doubled the odds (OR = 2.51; 95% CI: 1.79–3.54; *p* < 0.001). Moderate consumption of sugary drinks and juices was associated with 55% higher odds of having a less sustainable lifestyle (OR = 1.61; 95% CI: 1.41–1.84; *p* < 0.001), while these odds increased to 82% with daily consumption (OR = 1.82; 95% CI: 1.50–2.22; *p* < 0.001). Regarding the moderate consumption of salty snacks, 51% increased odds of having a low EVS score were observed (OR = 1.51; 95% CI: 1.32–1.73; *p* < 0.001). Furthermore, those who consumed sweet snacks moderately had 27% increased odds of having a poor EVS profile (OR = 1.27; 95% CI: 1.11–1.46; *p* < 0.001). This probability increased to almost 42% in excessive consumers of this UPF group (OR = 1.42; 95% CI: 1.20–1.67; *p* < 0.001) (Table 3).

## 4. Discussion

Unlike previous studies focusing exclusively on health outcomes, this study empirically links ultra-processed food consumption with multidimensional sustainable lifestyle behaviors across multiple countries. In this study, which examined the association between UPF consumption and sustainable lifestyles, it was found that participants with moderate to excessive UPF consumption were more likely to be in the least sustainable quartile compared to the most sustainable quartile.

Behaviorally, individuals with high UPF consumption may be more prone to impulsive food choices, lower environmental awareness and sedentary routine factors that contribute to unsustainable lifestyles. From an environmental perspective, UPFs are resource-intensive, relying heavily on energy, packaging and long-distance transportation, which increases their ecological footprint. These findings are aligned with previous studies highlighting the dual burden of UPFs: worsening population health and exacerbating climate and environmental damage [34,35].

The results of this study regarding BMI show that excessive consumption of UPF is associated with an increase in this parameter; in this case, the participants who exhibit this pattern were classified as overweight. Two prospective cohorts (n = 1827 and n = 8451) have demonstrated an approximately 20–30% higher risk of large increases in weight and waist circumference and a higher incidence of overweight/obesity. Participants in the highest quartile of ultra-processed food consumption had a higher risk of developing overweight or obesity than those in the lowest quartile [36,37].

We have also observed that the highest consumption of UPF was found in the young adult population, as observed in the studies by Andrade et al. and Silveira et al. [38,39]. We have also noticed that people with a higher educational level consumed them in lower proportions, a result supported by those obtained by Marrón-Ponce et al., in whose study it was confirmed that people with less education have a higher consumption of this type of food product [40]. The high consumption of UPF recorded in the younger population could be explained, in part, by their greater willingness to adopt new dietary patterns and innovations [41], as well as by the intense marketing strategy for industrialized products aimed at this age group [42].

It should be noted that trends in ultra-processed food consumption vary by country. However, it should be noted that the growing popularity of ultra-processed foods in Latin America is significantly associated with the prevalence of non-communicable diseases in this region. This association is gradual and reflects the growing urbanization and interaction with external markets in the Latin American economy [10].

Being over 60 years old, having a basic education level, and being female were determining factors for consuming fast food, sweet snacks, beverages and juices, with the exception of the latter, where men consumed them the most. Specifically, it was found that consumption of sugary drinks and juices is determined by sex (male), being over 40 years of age and having a primary education level, as seen in a previous study in 12 Latin American countries [43]. On the other hand, lower consumption of salty snacks was found among women, those under 60 years of age and those with a medium educational level. In this regard, a study found that gender, academic level and age are factors that influence the consumption of salty snacks. People with lower income and education and men consumed substantially more calories from these sources according to the study by Cohen et al. [44]. This may be because women consumed significantly fewer of these calories than men, considering their lower energy needs, and higher educational attainment and age may be considered strong predictors of lower total and relative calorie intake from these sources.

The main and most notable result we obtained in this study is that a higher consumption of UPF, in all its food groups (fast food, sugary drinks, sweet and salty snacks) was significantly associated with lower scores on the sustainable lifestyle index; that is, people with moderate to excessive consumption are more likely to be in the least sustainable quartile of lifestyles (Q1) than in the most sustainable (Q4). These findings indicate a clear inverse association between the frequency of UPF consumption and adherence to sustainable lifestyle behaviors in a large sample from Latin America and Spain. This supports the growing body of literature suggesting that dietary patterns high in UPFs are detrimental not only to individual health but also to environmental sustainability [45].

The fact that fast food and sugary drinks and juices were most likely to be in the least sustainable quartile is consistent with their well-documented environmental and health costs, including plastic packaging waste, water use, and links to obesity and metabolic diseases [46].

Reducing the intake of ultra-processed foods could yield substantial benefits for both individual health and planetary well-being. From a health perspective, lowering UPF consumption has been linked to reduced risks of obesity, metabolic syndrome and other non-communicable diseases. Such dietary shifts could help alleviate the growing burden on healthcare systems in the region. Environmentally, a population-level reduction in UPF demand could significantly decrease greenhouse gas emissions, plastic waste and the overexploitation of natural resources associated with industrial food production. It would also foster greater demand for locally produced, minimally processed foods, potentially supporting regional food sovereignty and biodiversity. Moreover, such changes could help realign food systems with sustainability targets outlined in global frameworks such as the UN Sustainable Development Goals (SDGs), particularly SDG 3 (Good Health and Well-being) and SDG 12 (Responsible Consumption and Production) [47].

In summary, our findings support the need to model food environments that guarantee food security, while ensuring food sovereignty and sustainable food systems, contextually appropriate and culturally accepted by each region for the benefit of health and the environment.

### Strengths and Limitations

The strengths of this study include its large, diverse sample across multiple countries, the use of a validated sustainability behavior index, and a focus on UPFs using the internationally recognized NOVA classification. This association may have a bidirectional behavior; that is, the consumption of UPFs may determine an unsustainable lifestyle and vice versa. With these findings, we offer the scientific community not only a starting point for determining the consumption of these foods from a nutritional point of view, but also a tool for evaluating another important aspect in people’s lives, such as sustainable lifestyles.

However, several limitations should be acknowledged. The cross-sectional design prevents causal inference, and the non-probabilistic sampling may limit generalizability. Data were self-reported, introducing potential recall and social desirability biases. The high proportion of female participants may limit generalizability, although similar patterns have been observed in population-based surveys [48,49,50]. Additionally, environmental context variables were not included, such as the availability of sustainable food options or national-level policies. In order to overcome these methodological limitations, it is suggested to conduct future prospective research that includes socioeconomic, income, employment, clinical and cultural variables that also have random sampling to obtain a representative sample according to the variables that we have exposed in this study.

## 5. Conclusions

This multicenter study shows that high UPF consumption is inversely associated with sustainable lifestyles. Our results reinforce the need to integrate environmental criteria into dietary guidelines and policy actions. Reducing UPF intake should be prioritized in sustainability and public health agendas, particularly in countries undergoing rapid nutrition transitions. These findings suggest that reducing UPF consumption could serve as a dual strategy to promote both healthier and more sustainable lifestyles, supporting integrated public health and environmental policies. The approach to sustainable lifestyles must be based on objective assessments of eating patterns and their impact on them, and with this, we are opening an emerging line of research in this area.

## Figures and Tables

**Table 1:** Sociodemographic and anthropometric characteristics of participants according to ultra-processed food consumption categories.

Consumption of Ultra-Processed Foods					
	Does Not Consume (n = 1134)	Moderate Consumption (n = 4563)	Excessive Consumption (n = 312)	Total (6009)	*p* *
**Sex (n-%)**					
Female	901 (79.5)	3603 (79.0)	211 (67.6)	4715 (78.5)	<0.001
Male	233 (20.5)	960 (21.0)	101 (32.4)	1294 (21.5)	
**Age**					
**Mean ± SD**	41.59 ± 14.38	33.86 ± 11.59	27.29 ± 9.04	34.98 ± 12.55	<0.001
**Age classification (n-%)**					
<30 years	280 (24.7)	1945 (42.6)	214 (68.6)	2439 (40.6)	<0.001
30–60 years	708 (62.4)	2478 (54.3)	95 (30.4)	3281 (54.6)	
>60 years	146 (12.9)	140 (3.1)	3 (1.0)	289 (4.8)	
**Education level (n-%)**					
Basic	98 (8.6)	589 (12.9)	62 (19.9)	749 (12.5)	<0.001
Medium	908 (80.1)	3446 (75.5)	204 (65.4)	4558 (75.9)	
Superior	128 (11.3)	528 (11.6)	46 (14.7)	702 (11.7)	
**Residence**					
Rural	136 (12.0)	584 (12.8)	58 (18.6)	778 (12.9)	0.007
Urban	998 (88.0)	3979 (87.2)	254 (81.4)	5231 (87.1)	
**BMI (kg/m^2^)**					
**Mean ± SD**	24.51 ± 4.46	25.45 ± 5.28	25.91 ± 6.23	25.3 0 ± 5.20	<0.001
**Country (n-%)**					
**Argentina**	186 (16.4)	535 (11.7)	18 (5.8)	739 (12.3)	<0.001
**Bolivia**	46 (4.1)	229 (5.0)	41 (13.1)	316 (5.3)	
**Chile**	149 (13.1)	582 (12.8)	23 (7.4)	754 (12.5)	
**Colombia**	124 (10.9)	426 (9.3)	11 (3.5)	561 (9.3)	
**Costa Rica**	57 (5.0)	355 (7.8)	15 (4.8)	427 (7.1)	
**Ecuador**	62 (5.5)	315 (6.9)	32 (10.3)	409 (6.8)	
**El Salvador**	14 (1.2)	300 (6.6)	61 (19.6)	375 (6.2)	
**Spain**	221 (19.5)	421 (9.2)	10 (3.2)	652 (10.9)	
**Guatemala**	16 (1.4)	202 (4.4)	7 (2.2)	225 (3.7)	
**Mexico**	76 (6.7)	414 (9.1)	20 (6.4)	510 (8.5)	
**Panama**	40 (3.5)	162 (3.6)	5 (1.6)	207 (3.4)	
**Paraguay**	27 (2.4)	245 (5.4)	18 (5.8)	290 (4.8)	
**Peru**	89 (7.8)	279 (6.1)	45 (14.4)	413 (6.9)	
**Uruguay**	27 (2.4)	98 (2.1)	6 (1.9)	131 (2.2)	
**Smoking**					
**No**	827 (72.9)	3485 (76.4)	245 (78.5)	4557 (75.8)	0.027
**Yes**	307 (27.1)	1078 (23.6)	67 (21.5)	1452 (24.2)	
**Physical activity**					
**No**	530 (46.7)	2560 (56.1)	201 (64.4)	3291 (54.8)	<0.001
**Yes**	604 (53.3)	2003 (43.9)	111 (35.6)	2718 (45.2)	

* Chi square was used for categorical variables, and ANOVA for continuous variables.

**Table 2:** Frequency of ultra-processed food consumption by sociodemographic characteristics.

Sociodemographic Characteristics									
UPF Groups	Educational Level			Age Range			Gender		
	Basic	Medium	Superior	<30 Years	30–60 Years	>60 Years	Female	Male	Total
**Fast food (n-%)**									
Does not consume	98 (13.1%)	908 (19.9%)	128 (18.2%)	280 (11.5%)	708 (21.6%)	146 (50.5%)	901 (19.1%)	233 (18.0%)	1134 (18.9%)
Moderate consumption	589 (78.6%)	3446 (75.6%)	528 (75.2%)	1945 (79.7%)	2478 (75.5%)	140 (48.4%)	3603 (76.4%)	960 (74.2%)	4563 (75.9%)
Excessive consumption	62 (8.3%)	204 (4.5%)	46 (6.6%)	214 (8.8%)	95 (2.9%)	3 (1.0%)	211 (4.5%)	101 (7.8%)	312 (5.2%)
* *p*- **value**			***<0.001***			***<0.001***		***<0.001***	
**Beverages and juices (n-%)**									
Does not consume	213 (28.4%)	1978 (43.4%)	270 (38.5%)	733 (30.1%)	1530 (46.6%)	198 (68.5%)	2020 (42.8%)	441 (34.1%)	2461 (41.0%)
Moderate consumption	347 (46.3%)	1901 (41.7%)	301 (42.9%)	1156 (47.4%)	1331 (40.6%)	62 (21.5%)	1987 (42.1%)	562 (43.4%)	2549 (42.4%)
Excessive consumption	189 (25.2%)	679 (14.9%)	131 (18.7%)	550 (22.6%)	420 (12.8%)	29 (10.0%)	708 (15.0%)	291 (22.5%)	999 (16.6%)
* *p*-value			**<*0.001***			**<*0.001***		**<*0.001***	
**Sweet snacks (n-%)**									
Does not consume	183 (24.4%)	1510 (33.1%)	220 (31.3%)	539 (22.1%)	1239 (37.8%)	135 (46.7%)	1512 (32.1%)	401 (31.0%)	1913 (31.8%)
Moderate consumption	347 (46.3%)	1917 (42.1%)	302 (43.0%)	1130 (46.3%)	1330 (40.5%)	106 (36.7%)	2015 (42.7%)	551 (42.6%)	2566 (42.7%)
Excessive consumption	219 (29.2%)	1131 (24.8%)	180 (25.6%)	770 (31.6%)	712 (21.7%)	48 (16.6%)	1188 (25.2%)	342 (26.4%)	1530 (25.5%)
* *p*-value			**<*0.001***			**<*0.001***		***0.612***	
**Salty snacks (n-%)**									
Does not consume	277 (37.0%)	2344 (51.4%)	327 (46.6%)	931 (38.2%)	1818 (55.4%)	199 (68.9%)	2374 (50.3%)	574 (44.4%)	2948 (49.1%)
Moderate consumption	339 (45.3%)	1735 (38.1%)	292 (41.6%)	1086 (44.5%)	1203 (36.7%)	77 (26.6%)	1836 (38.9%)	530 (41.0%)	2366 (39.4%)
Excessive consumption	133 (17.8%)	479 (10.5%)	83 (11.8%)	422 (17.3%)	260 (7.9%)	13 (4.5%)	505 (10.7%)	190 (14.7%)	695 (11.6%)
* *p*-value			**<*0.001***			**<*0.001***		**<*0.001***	

* Chi square.

**Table 3:** Association between ultra-processed food consumption and sustainable lifestyle scores.

Frequency of Consumption	Food and Shopping		Transport, Recreation and Self-Care		Environment		Total Score	
	OR, 95% CI	*p*-Value	OR, 95% CI	*p*-Value	OR, 95% CI	*p*-Value	OR, 95% CI	*p*-Value
**Fast food**								
Does not consume	Ref		Ref		Ref		Ref	
Moderate consumption	1.53 [1.31–1.78]	<0.001	1.69 [1.45–1.97]	<0.001	1.75 [1.51–2.03]	<0.001	1.97 [1.70–2.29]	<0.001
Excessive consumption	1.50 [1.09–2.06]	<0.001	1.43 [1.03–1.98]	0.034	1.92 [1.37–2.67]	0.034	2.51 [1.79–3.54]	<0.001
**Beverages and juices**								
Moderate consumption	1.18 [1.03–1.36]	0.017	1.43 [1.24–1.65]	<0.001	1.55 [1.35–1.78]	<0.001	1.61 [1.41–1.84]	<0.001
Excessive consumption	1.03 [0.85–1.24]	0.767	1.58 [1.28–1.93]	<0.001	1.59 [1.31–1.93]	<0.001	1.82 [1.50–2.22]	<0.001
**Salty snacks**								
Moderate consumption	1.43 [1.25–1.64]	<0.001	1.37 [1.19–1.57]	<0.001	1.51 [1.32–1.73]	<0.001	1.51 [1.32–1.73]	<0.001
Excessive consumption	1.16 [0.95–1.42]	0.155	1.22 [0.98–1.52]	0.079	1.14 [0.93–1.40]	0.215	1.23 [1.00–1.51]	0.051
**Sweet snacks**								
Moderate consumption	1.18 [1.02–1.36]	0.001	1.42 [1.23–1.65]	<0.001	1.40 [1.22–1.61]	<0.001	1.27 [1.11–1.46]	<0.001
Excessive consumption	1.28 [1.08–1.52]	<0.001	1.32 [1.11–1.56]	0.001	1.50 [1.27–1.77]	0.001	1.42 [1.20–1.67]	0.001

## Data Availability

The original contributions presented in this study are included in the article/Supplementary Materials. Further inquiries can be directed at the corresponding author.

## Supplementary Materials

The following supporting information can be downloaded at: https://www.mdpi.com/article/10.3390/nu18020365/s1. Table S1: UPF consumption of total population

## References

[1] Haththotuwa R.N., Wijeyaratne C.N. (2020). Worldwide epidemic of obesity. Obesity and Obstetrics.

[2] NCD Risk Factor Collaboration (NCD-RisC) (2024). Worldwide trends in underweight and obesity from 1990 to 2022: A pooled analysis of 3663 population-representative studies with 222 million children, adolescents, and adults. Lancet.

[3] Masood B., Moorthy M. (2023). Causes of obesity: A review. Clin. Med..

[4] Harb A.A., Shechter A., Koch P.A., St-Onge M.P. (2023). Ultra-processed foods and the development of obesity in adults. Eur. J. Clin. Nutr..

[5] Hall K.D. (2018). Did the Food Environment Cause the Obesity Epidemic?. Obesity.

[6] Monteiro C.A., Cannon G., Lawrence M., Costa Louzada M., Pereira Machado P. (2019). Ultra-Processed Foods, Diet Quality, and Health Using the NOVA Classification System.

[7] Monteiro C.A., Cannon G., Levy R.B., Moubarac J.-C., Louzada M.L.C., Rauber F., Khandpur N., Cediel G., Neri D., Martinez-Steele E. (2019). Ultra-processed foods: What they are and how to identify them. Public. Health Nutr..

[8] FAO, IFAD, UNICEF, WFP, WHO (2025). The State of Food Security and Nutrition in the World 2025—Addressing High Food Price Inflation for Food Security and Nutrition.

[9] Gómez-Donoso C., Martínez-González M.A., Bes-Rastrollo M. (2021). Disentangling nutrition facts from fiction: Towards healthy and sustainable consumption in industrialized societies. Metode Sci. Stud. J..

[10] Matos R.A., Adams M., Sabaté J. (2021). Review: The Consumption of Ultra-Processed Foods and Non-Communicable Diseases in Latin America. Front. Nutr..

[11] Why Sustainable Lifestyles Matter. https://www.unep.org/explore-topics/resource-efficiency/what-we-do/sustainable-lifestyles/why-sustainable-lifestyles.

[12] Macheka L., Kanter R., Lawrence M., Dernini S., Naja F., Oenema S. (2025). Sustainable diets: Where from and where to?. J. Nutr. Sci..

[13] Durán-Agüero S., Valdés-Badilla P., Valladares M., Espinoza V., Mena F., Oñate G., Fernandez M., Godoy-Cumillaf A., Crovetto M. (2023). Consumption of ultra-processed food and its association with obesity in Chilean university students: A multi-center study Ultra-processed food and obesity in Chilean university students. J. Am. Coll. Health.

[14] Zapata M.E., Arrieta E., Beltramo B., Rovirosa A. (2023). Ultra-processed food consumption in Argentina according to income level and its association with the intake of healthy foods. Nutr. Bull..

[15] Berthomier Rodríguez A.L., Duarte Amarilla N.J., Trinidad Rodríguez M.M., Núñez Martínez B.E., Meza-Miranda E.R. (2022). Processed and ultra-processed foods consumption in adults and its relationship with quality of life and quality of sleep. Rev. Nutr..

[16] Pereyra González I., Farías-Antúnez S., Buffarini R., Gómez Ayora A., Fletcher A.M., Rodrigues Domingues M., Freitas da Silveira M., Ferreira Umpiérrez A.H. (2023). Ultra-processed food consumption and the incidence of obesity in two cohorts of Latin-American young children: A longitudinal study. J. Pediatr. Nurs..

[17] Neri D., Steele E.M., Khandpur N., Cediel G., Zapata M.E., Rauber F., Marrón-Ponce J.A., Machado P., Louzada M.L.d.C., Andrade G.C. (2022). NOVA Multi-Country Study Group on Ultra-Processed Foods, Diet Quality and Human Health. Ultraprocessed food consumption and dietary nutrient profiles associated with obesity: A multicountry study of children and adolescents. Obes. Rev..

[18] Lane M.M., Gamage E., Du S., Ashtree D.N., McGuinness A.J., Gauci S., Baker P., Lawrence M., Rebholz C.M., Srour B. (2024). Ultra-processed food exposure and adverse health outcomes: Umbrella review of epidemiological meta-analyses. BMJ.

[19] Gajardo D., Gómez G., Carpio-Arias V., Landaeta-Díaz L., Ríos I., Parra S., Flores J.A.A., Illanes G.R.M., Meza E., Núñez B. (2025). Association between low dairy consumption and determinants of health in Latin American university students: A multicenter study. Nutr. Hosp..

[20] Morales G., Durán-Agüero S., Parra-Soto S., Landaeta-Díaz L., Carpio V., Cavagnari B., Rios-Castillo I., Nava-González E., Bejarano-Roncancio J., Núñez-Martínez B. (2023). Ultra-processed food and homemade fried food consumption is associated with overweight/obesity in Latin American university students during COVID-19. Am. J. Hum. Biol..

[21] Gering S.J., Martins C.A., Marques N.M.P., Cattafesta M., da Cunha A.C., Soares F.L.P., Santos Neto E.T.d., Salaroli L.B. (2024). The Consumption of Ultra-Processed Foods Is Associated with Abdominal Obesity in Individuals on Hemodialysis in Brazil. Obesities.

[22] Castrillón-Ruiz L., Estrada-Restrepo A., Cediel G., Cárdenas-Sánchez D., Barona-Acevedo J., Aristizábal J.C. (2025). Association between ultra-processed food intake, diet quality, and cardiovascular risk factors among adolescents in Antioquia, Colombia. Front. Public Health.

[23] Ordóñez Y., Saavedra-Clarke S., Reyes-García S., Crovetto M., Valladares M., Espinoza V., Machuca-Barria C., Cresp-Barria M., Durán-Agüero S. (2024). Diet and sleep quality in chilean university students. Int. J. Adolesc. Med. Health.

[24] Daas M.C., Vellinga R.E., Pinho M.G.M., Boer J.M.A., Verschuren W.M.M., van der Schouw Y.T., Van’t Veer P., Biesbroek S. (2024). The role of ultra-processed foods in plant-based diets: Associations with human health and environmental sustainability. Eur. J. Nutr..

[25] Seferidi P., Scrinis G., Huybrechts I., Woods J., Vineis P., Millett C. (2020). The neglected environmental impacts of ultra-processed foods. Lancet Planet. Health.

[26] de Araújo T.P., de Moraes M.M., Magalhães V., Afonso C., Santos C., Rodrigues S.S.P. (2021). Ultra-Processed Food Availability and Noncommunicable Diseases: A Systematic Review. Int. J. Environ. Res. Public Health.

[27] Cárcamo Vergara D.R., Salazar A.M., Cornejo V., Andrews M., Durán Agüero S., Leal Witt M.J. (2021). Alimentos ultraprocesados y su relación con la obesidad y otras enfermedades crónicas no transmisibles: Una revision sistemática. Rev. Esp. Nutr. Comunitaria.

[28] Lane M.M., Davis J.A., Beattie S., Gómez-Donoso C., Loughman A., O’Neil A., Jacka F., Berk M., Page R., Marx W. (2021). Ultraprocessed food and chronic noncommunicable diseases: A systematic review and meta-analysis of 43 observational studies. Obes. Rev..

[29] Anastasiou K., Baker P., Hadjikakou M., Hendrie G., Lawrence M. (2022). A conceptual framework for understanding the envi-ronmental impacts of ultra-processed foods and implications for sustainable food systems. J. Clean. Prod..

[30] Monteiro C.A., Cannon G., Moubarac J.C., Levy R.B., Louzada M.L.C., Jaime P.C. (2018). The UN Decade of Nutrition, the NOVA food classification and the trouble with ultra-processing. Public Health Nutr..

[31] Parra-Soto S., Carpio-Arias T.V., Rios-Castillo I., Pérez-Armijo P., Landaeta-Díaz L., Murillo A.G., Araneda-Flores J., Cavagnari B.M., Gómez G., Morales G. (2025). Dietary Patterns and Sustainable Lifestyles: A Multicenter Study from Latin America and Spain. Foods.

[32] World Health Organization Obesity: Preventing and Managing the Global Epidemic: Report of a WHO Consultation on Obesity, Geneva, 3–5 June 1997.

[33] Durán Agüero S., Valdés P., Godoy A., Herrera T. (2014). Hábitos alimentarios y condición física en estudiantes de pedagogía en educación física. Rev. Chil. Nutr..

[34] Springmann M., Clark M.A., Rayner M., Scarborough P., Webb P. (2021). The global and regional costs of healthy and sustainable dietary patterns: A modelling study. Lancet Planet. Health.

[35] Alsaffar A.A. (2016). Sustainable diets: The interaction between food industry, nutrition, health and the environment. Food Sci. Technol. Int..

[36] Canhada S.L., Luft V.C., Giatti L., Duncan B.B., Chor D., Fonseca M.J.M.D., Matos S.M.A., Molina M.D.C.B., Barreto S.M., Levy R.B. (2020). Ultra-processed foods, incident overweight and obesity, and longitudinal changes in weight and waist circumference: The Brazilian Longitudinal Study of Adult Health (ELSA-Brasil). Public Health Nutr..

[37] Mendonça R.D., Pimenta A.M., Gea A., de la Fuente-Arrillaga C., Martinez-Gonzalez M.A., Lopes A.C., Bes-Rastrollo M. (2016). Ultraprocessed food consumption and risk of overweight and obesity: The University of Navarra Follow-Up (SUN) cohort study. Am. J. Clin. Nutr..

[38] Calixto Andrade G., Julia C., Deschamps V., Srour B., Hercberg S., Kesse-Guyot E., Allès B., Chazelas E., Deschasaux M., Touvier M. (2021). Consumption of Ultra-Processed Food and Its Association with Sociodem-ographic Characteristics and Diet Quality in a Representative Sample of French Adults. Nutrients.

[39] Silveira V.N.C., Dos Santos A.M., França A.K.T.C. (2024). Determinants of the consumption of ultra-processed foods in the Brazilian population. Br. J. Nutr..

[40] Marrón-Ponce J.A., Sánchez-Pimienta T.G., Louzada M.L.D.C., Batis C. (2018). Energy contribution of NOVA food groups and so-ciodemographic determinants of ultra-processed food consumption in the Mexican population. Public Health Nutr..

[41] L’Anses A.D. Étude Individuelle Nationale des Consommations Alimentaires 3 (INCA 3) Paris, France: 2017. https://www.anses.fr/fr/content/inca-3-evolution-des-habitudes-et-modes-de-consommation-de-nouveaux-enjeux-en-mati%C3%A8re-de.

[42] Lobstein T., Dibb S. (2005). Evidence of a possible link between obesogenic food advertising and child overweight. Obes. Rev..

[43] Meza-Miranda E., Núñez-Martínez B., Durán-Agüero S., Pérez-Armijo P., Martin-Cavagnari B., Cordón-Arrivillaga K., Carpio-Arias V., Nava-González E.J., Camacho-López S., Ivankovich-Guilén S. (2021). Consumo de Bebidas azucaradas durante la pandemia por Covid-19 en doce países iberoamericanos: Un estudio transversal. Rev. Chil. Nutr..

[44] Cohen D.A., Sturm R., Scott M., Farley T.A., Bluthenthal R. (2010). Not enough fruit and vegetables or too many cookies, candies, salty snacks, and soft drinks?. Public Health Rep..

[45] Fardet A., Rock E. (2020). Ultra-Processed Foods and Food System Sustainability: What Are the Links?. Sustainability.

[46] Serafini M., Toti E. (2016). Unsustainability of Obesity: Metabolic Food Waste. Front. Nutr..

[47] Northcott T., Lawrence M., Parker C., Reeve B., Baker P. (2025). Regulatory responses to ultra-processed foods are skewed towards behaviour change and not food system transformation. Nat. Food.

[48] Ruani M.A., Reiss M.J., Kalea A.Z. (2023). Diet-Nutrition Information Seeking, Source Trustworthiness, and Eating Behavior Changes: An International Web-Based Survey. Nutrients.

[49] Colby S., Zhou W., Allison C., Mathews A.E., Olfert M.D., Morrell J.S., Byrd-Bredbenner C., Greene G., Brown O., Kattelmann K. (2020). Development and Validation of the Short Healthy Eating Index Survey with a College Population to Assess Dietary Quality and Intake. Nutrients.

[50] Henriksen H.B., Knudsen M.D., Hjartåker A., Blomhoff R., Carlsen M.H. (2024). Digital Food Frequency Questionnaire Assessing Adherence to the Norwegian Food–Based Dietary Guidelines and Other National Lifestyle Recommendations: Instrument Validation Study. J. Med. Internet Res..

