# Supplementary material for: Consumption of Ultra-Processed Foods and Sustainable Lifestyles: A Multicenter Study
Source: Nutrients. 2026 Jan 22;18(2):365. doi: 10.3390/nu18020365 (PMC12845370; doi:10.3390/nu18020365)
Supplement: Supplementary file 1 [file nutrients-18-00365-s001.zip › nutrients-4079221-supplementary.pdf]

Supplementary:

Supplementary Table S1: **UPF consumption of total population**

**Fast-food (n-%)**	
Does not consume	894 (14.8%)
Moderate consumption	3433 (56.7%)
Excessive consumption	1725 (28.5%)
**Beverages and juices (n-%)**	
Does not consume	2483 (41.0%)
Moderate consumption	2562 (42.3%)
Excessive consumption	1007 (16.6%)
**Sweet snacks (n-%)**	
Does not consume	1933 (31.9%)
Moderate consumption	2582 (42.7%)
Excessive consumption	1537 (25.4%)
**Salty snacks (n-%)**	
Does not consume	2974 (49.1%)
Moderate consumption	2382 (39.4%)
Excessive consumption	696 (11.5%)
